# Can Moxidectin Be an Anthelmintic Alternative for Trichuris trichiura and Strongyloides stercoralis: A Systematic Review

**DOI:** 10.7759/cureus.27074

**Published:** 2022-07-20

**Authors:** Stephanie P Fabara, Ghanshyam Patel, Nidhi Jain, Daniel Bishev, Belen Tama, Angelo Caputi, Daniel Zarrate, Jaffar A Al-Tawfiq, Raghavendra Tirupathi

**Affiliations:** 1 Internal Medicine, North Florida Regional Medical Center, Gainesville, USA; 2 Intenal Medicine, Mercy Health - Javon Bea Hospital, Rockford, USA; 3 Medicine and Surgery, Himalayan Institute of Medical Sciences, Dehradun, IND; 4 Hematology and Oncology, Brooklyn Cancer Care, Brooklyn, USA; 5 Internal Medicine, Sir Ganga Ram Hospital, New Delhi, IND; 6 Internal Medicine, Nassau University Medical Center, East Meadow, USA; 7 Medical School, American University of the Caribbean School of Medicine, Cupecoy, SXM; 8 Medicine, Universidad Católica de Santiago de Guayaquil, Guayaquil, ECU; 9 General Medicine, Universidad Catolica de Santiago de Guayaquil, Guayaquil, ECU; 10 General Medicine, El Bosque University, Bogota, COL; 11 Internal Medicine, Johns Hopkins Aramco Healthcare, Dhahran, SAU; 12 School of Medicine, Johns Hopkins University, Baltimore, USA; 13 Internal Medicine, Keystone Health, Chambersburg, USA

**Keywords:** soil microbes, strongyloides stercoralis, trichuris trichiura, anthelminthic, moxidectin

## Abstract

Strongyloides stercoralis and Trichuris trichiura parasitic infections are two of the many neglected tropical diseases. These parasitic infections are of considerable public health relevance, particularly in resource-limited countries. Moxidectin, a well-established drug in veterinary medicine, is now a Food and Drug Administration (FDA) approved medication for human onchocerciasis. For the past five years, this medication has been under clinical trials to evaluate its efficacy and safetiness in other helminthic infections. Moxidectin might complement the already existing treatment and control of soil-transmitted helminthiasis (STH). Therefore, we systematically reviewed existing human interventional studies to evaluate the efficacy and safety of this medication when administered alone or in combination with other antiparasitic medications in order to achieve a cure.

## Introduction and background

Infections with soil-transmitted helminths (STH) are most common in people living in or coming from areas with poor access to adequate water, sanitation, and hygiene [1]. *Strongyloides stercoralis* causes the human disease Strongyloidiasis. Strongyloides is known to exist on all continents except Antarctica, but it is most common in the tropics, subtropics, and in warm temperate regions. The global prevalence is estimated to be between 30 and 100 million infected people worldwide [2]. Infections range from asymptomatic or subclinical infections to symptomatic strongyloidiasis and to the potentially life-threatening hyperinfection syndrome in immunocompromised patients [3]. The medical importance of strongyloidiasis resides in its capacity to remain clinically asymptomatic and chronically unnoticed until the host suffers alterations in its immune equilibrium that allow for accelerated larval reproduction that can lead to dissemination. *Trichuris trichiura* infection occurs worldwide, with the highest infection prevalence and intensity in children. In 2002, the estimated number of people infected with *T. trichiura* was 1 billion. Trichuriasis also occurs in the southern United States [4]. *T. trichiura* infections lack symptoms; only patients with heavy infections are symptomatic [5].

Moxidectin is a macrocyclic lactone, a member of the milbemycin family, which was developed in the 1980s and is widely used in veterinary medicine for heartworm, whipworm, and lungworm infections [6]. The success of moxidectin relies on a broad spectrum of activity, including efficacy against benzimidazole-resistant strains of helminths. The primary objective of the study is to assess the efficacy and safety of moxidectin alone and in co-administration against *T. trichiura* and *S. stercoralis* infection.

*T. trichiura *life cycle

According to the CDC, the *T. trichiura* life cycle [7] is described as the following: (1) passage of embryonated eggs in human stools, (2) development of eggs in the soil to two cell-stage, (3) stage of advanced cleavage, (4) eggs embryonate, (5) infective stage develops in 15-30 days, (6) on reaching the small intestine post-ingestion (through soil-contaminated food/hands), the eggs hatch and larvae are released, (7) larvae mature into adults in the large intestine, with adult worms (approx. 4 cm long) residing in the ascending colon and cecum, and (8) females oviposit 60-70 days post-infection shedding 3-20 thousand eggs every day in the cecum. *T. trichiura’s* life span of an adult worm is 1 year.

*S. stercoralis *life cycle

According to the CDC, it has a complicated life cycle that involves autoinfection and alternates between free-living and parasitic cycles [8].

Free-living cycle: (1) the infected definitive host passes the rhabditiform larvae in the stool, (2) it then develops into either infective filariform larvae (direct development) or free-living adult worms (male and female), (3) eggs are produced by fertilized female worms, (4) rhabditiform larvae hatch from embryonated eggs, and (5) the larvae eventually become infective filariform (L3) larvae.

Parasitic cycle: (6) the parasite cycle begins when filariform larvae penetrate the skin of the human host when skin contacts soil. (7) The filariform larvae migrate to the small intestine where they become adults. This second generation of filariform larvae is unable to grow into independent adults and must find a new host to continue the life cycle. (8) The L3 larvae migrate via the bloodstream and lymphatics to the lungs, where they are eventually coughed up and swallowed. It also migrates to the intestine via alternate routes like abdominal viscera or connective tissue. (9) In the small intestine, the larvae molt twice and become adult female worms. (10) The females live embedded in the submucosa of the small intestine and produce eggs via parthenogenesis (parasitic males do not exist). (11) Rhabditiform larvae hatch and can either be passed in the stool or migrate to the intestinal lumen, causing autoinfection by penetrating either the intestinal mucosa or the skin of the perianal area. (12) After reinfecting the host, the larvae are carried to the lungs, pharynx, and small intestine ending up disseminating throughout the body. If left untreated, a persistent infection develops, and even after many decades of residing in a non-endemic area, this would contribute to the development of hyperinfection syndrome.

Transmission

Infection with geohelminths is prevalent in regions where access to sanitation, hygiene, and clean water is poor. *T. trichiura* transmission occurs through the fecal-oral route, with ingestion of embryonated eggs via hands or food. On the other hand, *S. stercoralis* may be transmitted through multiple routes, including percutaneously (through skin penetration, particularly feet) and orally [1].

A Spanish study discovered the relevance of cultural and physical factors with respect to intestinal parasitosis. It also found the frequency of enteroparasitosis to be highest in children aged 3-8 years, especially in those living in households with overcrowding and using common outdoor latrines [9]. Another study conducted in Africa revealed the role of cockroaches as a reservoir for infectious pathogens, including ova of *T. trichiura*, thereby emphasizing the importance of controlling cockroaches to substantially minimize disease spread in our environment [10]. Khan et al. concluded that food handlers play a major role in the transmission of intestinal parasites and that poor education level, large family size as well as low family income are associated with an increased prevalence of parasitic infections [11].

Clinical manifestations

Trichuriasis is usually asymptomatic. Symptomatic individuals may present with diarrhea, abdominal pain, and asthenia, while serious cases manifest trichuris-dysenteric syndrome, presenting with abdominal tenderness, digital clubbing, rectal prolapse, and anemia (which may be severe). During a heavy infestation, rectal prolapse may occur. Children can manifest anemia, impaired cognitive development, and growth deficiency due to poor nutrition and iron deficiency owing to worm burden [12].

Strongyloidiasis is also an asymptomatic infection in those who are otherwise healthy. In these cases, peripheral eosinophilia may be the only infection sign. An itchy serpiginous skin rash, called ground itch, may follow acute infection in the area where larval penetration into the skin occurred (usually feet, but maybe perianal, hands, abdomen, etc.). Intradermal migration occurs quickly (5-15 cm/hour), leading to a rash known as larva currens. However, migration of larvae to the lungs may cause eosinophilic pneumonia, a Loeffler-like syndrome with wheezing, dyspnoea, dry cough, hemoptysis, migratory pulmonary infiltrates, and eosinophilia. During hyperinfection, pulmonary manifestations can become fatal [13]. Patients with chronic infections may complain of nausea, diarrhea, anorexia, abdominal pain and tenderness, and asthenia. Larva currens are a common manifestation of chronic infection, presenting as serpiginous urticaria, characteristically over the torso, abdomen, groin, and buttocks. Lesions may last for days and may recur weeks or months later without treatment. Rare immune-mediated diseases like reactive arthritis can also occur [1]. Although autoinfection produces negligible symptoms in immunocompetent individuals, in immunocompromised individuals it causes strongyloides-hyperinfection syndrome, occurring decades post-the first infection. Strongyloides-hyperinfection syndrome manifests as a pulmonary or intestinal failure with pronounced intestinal and cutaneous mucosal bleeding. If untreated, it has a nearly 100% mortality ratio. It occurs from accelerated, uncontrolled autoinfection leading to widespread dissemination of larvae to end organs, including musculoskeletal, hepatic, cardiovascular, urogenital, and central nervous systems. A common complication of disseminated strongyloidiasis is sepsis, which stems from bacterial translocation through the intestinal wall. Individuals at high risk include immunosuppressed patients, like those on immunosuppressive medications, especially vincristine and corticosteroids, as well as those with hematological malignancies, human T-cell lymphotropic virus type 1 infection, and hypogammaglobulinemia [13].

Materials and methods

Eligibility Criteria

Inclusion criteria were randomized controlled trials with active parasitic infection of *S. stercoralis* or *T. trichiura* in adolescents or adults, confirmed by stool samples treated with the aforementioned drug, while the exclusion criteria were *Onchocerca volvulus* parasitic infection.

Search Strategy

For this review, we used the Preferred Reporting Items for Systematic Reviews and Meta-Analyses (PRISMA) statement. All articles were retrieved from Medline, clinicaltrials.gov, and the Cochrane library database using the search term "moxidectin" for the topic. We also used an advanced search strategy with the following term: (moxidectin [Title/Abstract]). For further screening and selection, only full-text articles in English literature were considered to be included. A literature search was conducted up to June 2022 to identify all potential articles in the data source. In the screening process, research before 2012 was excluded for clinical relevance. After initial processing, all authors reviewed potentially eligible studies for exclusion and inclusion. Articles with animal studies, poor study design, studies other than clinical trials, and those that did not fulfill the study's outcome were considered not eligible.

Outcome Measures

Our primary outcome measure is the cure rate. The secondary outcomes are efficacy and safety.

Bias Assessment

For assessing bias, we used the Cochrane collaboration's tool risk assessment of clinical trials [14].

## Review

Results

Study Selection

**Figure 1:**
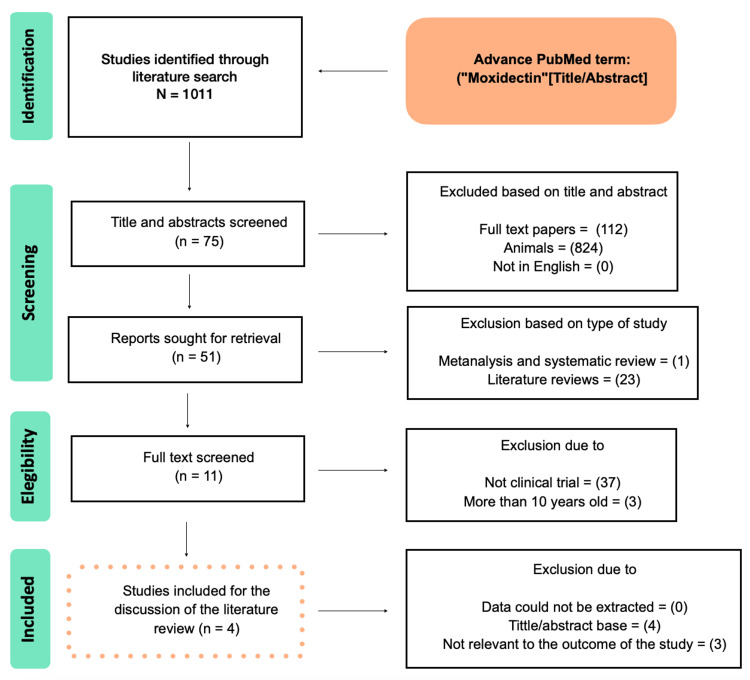
Shows the results of the study using a PRISMA flow chart.

A flowchart of article selection is shown in Figure 1 to illustrate the processes of database identification, article screening, and final inclusion according to the PRISMA statement. Using the search term and strategy mentioned above, we found 1,011 Medline articles on the topic. After the screening process to exclude articles based on title and abstract, type of study, not conducted in humans, other studies than clinical trials, and research before 2011, 12 articles were considered for further analysis. Each article was evaluated and extracted independently by all authors to determine the sources, including year, authors, research design, and clinical outcomes. After excluding articles with poor study design, data that could not be extracted, or that did not match our outcomes, there were four eligible articles for the topic.

Eligibility Criteria

Four studies were eligible in which the intervention was moxidectin vs. placebo or in combination with other anthelmintics for at least 28 days in patients infected with *S. stercoralis* or *T. trichiura*. All were randomized controlled clinical trials. Follow-up periods between studies were the same. All authors found differences within each intervention regarding dose, combination, and time of duration. We excluded all studies in which moxidectin was used for onchocerciasis.

Study Characteristics and Findings

The main features of the included studies for this review are shown in Table 1.

**Table 1:** Summary of studies included in this systematic review.

Author, year of the publication	Country	Study design	Study population	Number of patients in each group	Duration of treatment	Outcome measures	Findings
Barda et al. [15]	Laos	Exploratory, phase II, randomized, single-blind trial	Participants aged 12–60 years old with *S. stercoralis* infection	- Ivermectin = 62 - Moxidectin = 63	21 days	Cure rate	CR of 93.7% (59/63) for moxidectin compared to 95.2% (59/62) for ivermectin. Moxidectin might be a safe and efficacious alternative to ivermectin. Non-inferiority could not be demonstrated.
Barda et al. [6]	Tanzania	Randomized, single-blind, non-inferiority trial	Adolescents aged 12–18 years with *T. trichiura* infection	- Moxidectin + albendazole = 197 - Albendazole + oxantel pamoate = 200 - Moxidectin + tribendimidine = 119 - Moxidectin = 118	14–21 days	Cure rate	CR of 51% in moxidectin + albendazole, 83% in albendazole + oxantel pamoate, 23% in moxidectin + tribendimidine and 14% in moxidectin alone. Albendazole + oxantel pamoate showed a considerably higher cure rate.
Keller et al. [16]	Tanzania	Phase II, randomized, placebo-controlled, dose-finding trial	Adolescents 16–18 years old with *T. trichiura* infection	- 8 mg moxidectin = 41 - 16 mg moxidectin = 41 - 24 mg moxidectin = 42 - 8 mg moxidectin + 400 mg albendazole = 41 - 16mg moxidectin + 400 mg albendazole = 41 - 24 mg moxidectin + 400 mg albendazole= 42 - Placebo = 42	13–20 days	Cure rate	CRs against *T. trichiura* were 43, 46, and 44% for 8, 16, and 24 mg of moxidectin alone, respectively; 60, 62, and 66% for the same moxidectin dosages plus 400 mg of albendazole, respectively; and 12% for placebo
Hofmann et al. [17]	Laos	Randomized, parallel-group, single-blinded, placebo-controlled, dose-ranging, phase IIa trial	Adults aged 18–65 years with *S. stercoralis* infection	- 2 mg moxidectin = 30 - 4 mg moxidectin = 29 - 6 mg of moxidectin = 32 - 8 mg of moxidectin = 29 - 10 mg of moxidectin = 30 - 12 mg of moxidectin = 30 - Placebo = 29	28 days	Cure rate	CR of 14% in placebo, 75% in 2 mg, 83% in 4 mg, 86% in 6 mg, 87% in 8 mg, 88% 10 mg, and 88% in 12 mg. 4-12 mg of moxidectin showed promising tolerability and efficacy profiles in the treatment of *S. stercoralis* infections in adults

Assessment of Risk of Bias

The bias risk assessment of the included studies for this review is shown in Table 2.

**Figure 2:**
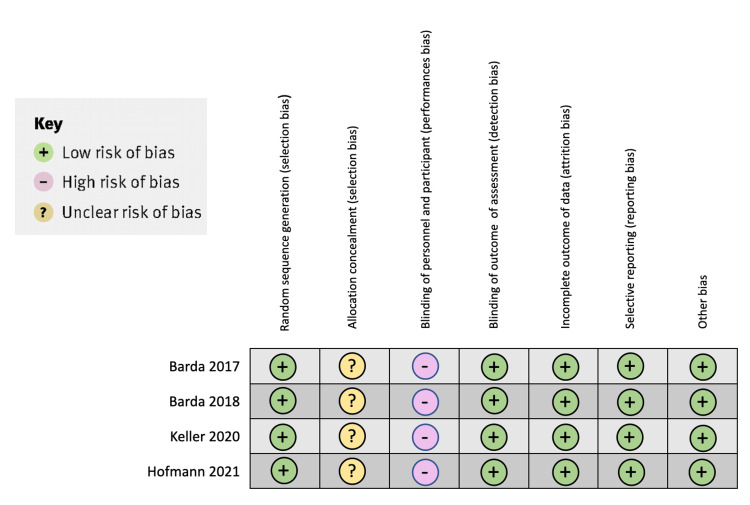
Shows the bias analysis of the clinical trials in the systematic review.

Participants Recruitment

Barda et al. recruited a sample size of 127 patients no younger than 12 years old from the villages of Morphu and Phakpheo in the province of Champasack, Laos. From each participant, two stool samples for two consecutive days were collected and tested with the Baermann method according to the standard WHO procedure for *S. stercoralis* larvae [15]. Similarly, Barda et al. recruited participants aged 12-18 years from two primary schools and one secondary school in Mkanyageni and Mchokocho, Tanzania. Two stool samples were provided by each participant, and those who tested positive for *T. trichiura* infection according to the Kato-Katz method were considered for inclusion [6]. In a similar fashion, Keller et al. recruited high school adolescents aged 16 to 18 in the Mkoani district, Tanzania. In order to be eligible, they must provide two stool samples positive for *T. trichiura* with at least 100 eggs per gram (EPG) on Kato-Katz thick smears, which were later dropped due to unexpectedly low infection intensities [16]. Whereas Hofmann et al. recruited adults aged 18-65 years. The enrollees were eligible if they were infected with *S. stercoralis* with an infection intensity of 0.4 larvae per gram of stool in at least two stool samples collected on different days based on Baermann assays [17].

In all the above-mentioned studies, the participants were excluded if on the initial clinical assessment they were found to have acute or uncontrolled systemic illness, a history of severe chronic diseases, had a positive pregnancy test, were breastfeeding, or if they were part of other clinical trials.

Patients Baseline Characteristics

According to Barda et al., baseline characteristics in both arms were well balanced, with a median age of 40, 51% male sex, a mean weight of 54 kg, and a mean height of 158 cm. The higher number of coinfections of *S. stercoralis* with *Opisthorchis viverrini* and hook worm was observed in the moxidectin arm compared to the ivermectin arm (89.1% and 58% vs. 75% and 56%, respectively) [15]. On the other hand, in the study by Barda et al., all four groups of participants were equally distributed with a mean age of 14 years, 57% were of female sex, and with a median of 534 *T. trichiura* EPG. Overall, 42% and 58% of participants were coinfected with hookworm and *Ascaris lumbricoides*, respectively, at baseline [6]. In the study by Keller et al., all treatment group participants were well balanced in their demographic characteristics and *T. trichiura* baseline infection intensity. The mean age of participants was 16.8 years [16]. The 209 participants in the Hofmann et al. study had similar baseline demographics and parasitological characteristics in all seven treatment groups. At baseline, 85% of participants had moderate to severe intensity *S. stercoralis* infections and the rest had low intensity infections. The mean age of participants was 43.8 years, 66% were male, and 63% and 67% had coinfection with hookworms and *O. viverrini*, respectively [17].

Interventions

Barda et al. screened 571 participants, of which 283 did not provide enough stool samples for screening, 153 were negative for infection, and 8 did not meet the inclusion criteria. Finally, a total of 127 participants were enrolled and randomly allocated to the two treatment arms for 21 days [15]: (1) 64 participants received a single dose of moxidectin 8 mg, and (2) 63 participants received a single dose of ivermectin 200 µg/kg.

In the study by Barda et al., a total of 942 participants were assessed for eligibility. After exclusion, 701 were randomly assigned to receive one out of four treatments (5:5:3:3) for 14-21 days [6]: (1) moxidectin 8 mg plus albendazole 400 mg arm: assigned 219, analyzed 197, (2) albendazole 400 mg plus oxantel pamoate 25 mg/kg arm: assigned 220, analyzed 200, (3) moxidectin 8 mg plus tribendimidine 200-400 mg arm: assigned 130, analyzed 119, and (4) moxidectin 8 mg arm: assigned 132, analyzed 118.

Keller et al. initially recruited 379 participants who were screened for *T. trichiura*. After screening and exclusion, a total of 290 adolescents were randomly assigned (41 or 42 participants per arm) to receive one out of seven treatment arms (1:1:1:1:1:1:1) for 14 to 21 days [16]: (1) 8, 16, or 24 mg of moxidectin alone, (2) 8, 16, or 24 mg of moxidectin plus 400 mg of albendazole, and (3) placebo.

Hofmann et al. first screened 785 individuals for acceptance, of whom 419 were negative for S. stercoralis and 122 had inadequate infection intensity for *S. stercoralis*. The remaining 223 adults were randomly assigned using computer-generated randomization to seven treatment groups, including placebo (1:1:1:1:1:1:1). In each arm, 30-33 were assigned, but 29-32 were analyzed [17]. Intervention was with moxidectin 2 mg, 4 mg, 6 mg, 8 mg, 10 mg, 12 mg, and placebo.

Limitations of the Clinical Trials

In the study by Barda et al., cure rates were lower, the study was underpowered, and non-inferiority could not be demonstrated at the pre-specified margin [15]. While the limitations of the study by Barda et al. suggest a better design, a double-blind trial would be impossible because the study included one weight-dependent (oxantel pamoate) and one age-dependent (tribendimidine) treatment group. Another limitation was that hookworm infections on Pemba Island are mainly mild, so only a few moderate or heavy infections were registered, making it difficult to accurately assess the efficacy against moderate and high intensities of hookworm infection [6]. In the study by Keller et al., participants and research staff were not blinded. Most of the patients with *T. trichiura* infections found in this study were mild, hence affecting the proportion of cured participants. Moreover, the efficacy and safety were only assessed up to 13-20 days post-treatment. Moxidectin has a long half-life, and the authors recommended that studies with extended follow-up periods should be conducted [16]. On the other hand, Hofmann et al. identified as one of their limitations the short follow-up period of 28 days (as per the WHO guidelines), as moxidectin has an elimination half-life of as long as 20-35 days. Therefore, they recommended trials with longer follow-ups. Another limitation is that diagnosing low-intensity infections with *S. stercoralis* is a challenge for its quantitative or qualitative analysis. The authors set a cutoff of at least 0.4 larvae per gram of stool in at least two stool samples, resulting in a diagnostic sensitivity of more than 97%, and to further increase diagnostic sensitivity, three stool samples were collected, if possible, on three consecutive days, and from each sample, two assays were prepared [17].

Discussion

For the present study, our primary outcome analyzed is the cure rate, and the secondary outcome is efficacy and safety of moxidectin in the treatment of *T. trichiura* and *S. stercoralis* alone or in combination with other antiparasitic drugs like ivermectin, albendazole, oxantel pamoate, and tribendimidine.

Efficacy and Safety Against S. stercoralis

Both drugs (moxidectin and ivermectin) tested by Barda et al. showed high efficacy against *S. stercoralis* infection. Moxidectin achieved a CR of 93.6% (59/63) (CI 84.5-98.2) compared to ivermectin, which showed a CR of 95.1% (59/62) (CI 86.5-99.0) [15]. Meanwhile, Hofmann et al. tested only moxidectin but with ascending doses in each group, showing a CR against *S. stercoralis* of 73% in the 2 mg group, 90% in 4 mg, 84% in 6 mg, 83% in 8 mg, 97% in 10 mg, and 87% in the 12 mg group. Therefore, increasing the dose translated into slightly higher efficacies that flattened at 8 mg. The placebo group had a CR of 14%. Observed larvae reduction rates against *S. stercoralis* were above 98% among all moxidectin treatment groups [17].

For moxidectin’s safety against *S. stercoralis*, Barda et al. showed that at clinical examination, 37 (29.1%) of subjects reported symptoms before treatment. Most of the participants had vertigo (13.4%) and headaches (8.6%). In addition, a few participants reported nausea, diarrhea, and abdominal discomfort with skin lesions, whereas one adult reported blood in the stool. Subjects were checked at different time points for side effects: 3, 24, and 48 hours after the administration of drugs. None of the participants reported any side effects of the treatment at any time point [15]. Hofmann et al. described moxidectin as well tolerated. There were no serious adverse events during the study period across all treatment groups. At baseline, 38/180 participants (21%) in moxidectin treatment groups reported having at least one symptom, with headache, pruritus, and diarrhea being the most commonly reported adverse events. After 3 and 24 hours of treatment, only ten (6%) and three (2%) of these adults, respectively, reported any symptoms. However, in the placebo group, seven (28%) participants reported symptoms after 3 hours of treatment and two (7%) participants reported symptoms 24 hours after treatment. At follow-up at 28 days, the total number of symptoms in the moxidectin groups was 33% of that at baseline [17].

Efficacy and Safety Against T. trichiura

Barda et al. showed that the co-administration of moxidectin plus albendazole was not inferior to the most efficacious co-administration, albendazole plus oxantel pamoate [6]. The moxidectin plus albendazole group had a significantly lower CR than the albendazole plus oxantel pamoate (CR 50.8% vs 83.0%, OR 4.7, 95% CI 3.0-7.6, p<0.0001) but significantly higher than moxidectin monotherapy (CR 14.4%, OR 6.1, 95% CI 3.5-11.3, p<0.0001). Moxidectin plus albendazole was superior to moxidectin monotherapy. CRs from light and moderate infections were 64% and 23%, respectively, for moxidectin plus albendazole, 29% and 9%, respectively, for moxidectin plus tribendimidine, and 20% and 3%, respectively, for moxidectin alone. None of the high-intensity infections of *T. trichiura* were cured by any treatment. The moxidectin monotherapy group failed to establish the non-inferiority margin against the moxidectin plus albendazole group, showing an ERR of 83.2 % and a CR of 14.4%. On the other hand, Keller et al. assessed moxidectin’s efficacy by the slight increase in CR and ERR at higher doses of moxidectin combined with albendazole [16]. CRs against *T. trichiura* were 45-50% in the moxidectin monotherapy group compared to 65-70% in the combination moxidectin-albendazole group.

In regards to safety, Barda et al. reported no serious adverse events during the study. All were mild and did not require any intervention [6]. Before the treatment, 66 (10%) of 634 participants reported mainly stomach pain and headaches. After the administration of all treatments, the adverse events were reported at 3, 24, and 48 hours. At 3 hours mild symptoms were reported (5-12%), at 24 hours the highest percentage of adverse events were reported (18-24%), and at 48 hours mild symptoms accounted for less than 10% in all arms. Stomach pain, constipation, and headache are the most frequently reported symptoms. Meanwhile, the safety evaluation by Keller et al. showed that 50 participants reported symptoms before the treatment; the most commonly reported adverse events before and after the treatment were abdominal pain and headache [16]. After treatment at 3, 24, 48, and 72-hour time points, the reported adverse events were 18, 13, 8, and 5%, respectively, without any difference in events among the treatment arms.

Based on the latest evidence, we, therefore, present recommendations for using this newly approved medication in the treatment of *T. trichura* and *S. stercoralis* infections.

Future Approach

Since moxidectin is already FDA approved for the treatment of O. volvulus, with this study we encourage future clinical trials to further explore this drug. For many years, it has been shown to be efficacious in the veterinary field. Therefore, implementing this medication with an established dose could be helpful in correctly treating these infections.

Study Limitations

The main limitation of this systematic review was the small number of published reports available on the efficacy and safety of moxidectin. The low number of eligible studies also prevented us from evaluating the possibility of publication bias. Therefore, our findings need to be confirmed by high-quality research studies with a rigorous design (e.g., double-blinded RCTs). The findings, while limited by the small number of studies and the lack of blinding, add to the evidence supporting the safety of moxidectin at 4-12 mg, which demonstrated overall good safety.

## Conclusions

In conclusion, study results showed that moxidectin might be a safe and efficacious alternative to ivermectin for the treatment of *S. stercoralis* infection since 4-12 mg of moxidectin showed promising tolerability and efficacy profiles in the treatment of infections in adults. However, in one *T. trichiura* trial, moxidectin-albendazole was shown to be superior to moxidectin, highlighting that there is no benefit to using doses above 8 mg, which is the recommended dose for onchocerciasis. The moxidectin-albendazole combination of 8 mg plus 400 mg should be further investigated. In the other trial, moxidectin plus albendazole showed non-inferiority to albendazole plus oxantel pamoate in terms of ERR; however, albendazole plus oxantel pamoate showed a considerably higher *T. trichiura* cure rate. The authors recommended dose-optimization studies with moxidectin and moxidectin plus albendazole since the 8 mg dose recommended for onchocerciasis might not be optimal for the treatment of *T. trichiura* infections.
